# Prevention of Malaria in Pregnancy: What Do the Pregnant Women of Nigeria Know and Do about It?

**DOI:** 10.1155/2022/7061548

**Published:** 2022-11-18

**Authors:** Stephen Kalu, Laurent Cleenewerck, Kabiru AbuBakar Gulma, Devender Bhalla

**Affiliations:** Pôle Universitaire Euclide Intergovernmental UN Treaty 49006/49007, Bangui, Central African Republic

## Abstract

**Objective:**

We assessed knowledge, attitude, and practice regarding two malaria prevention measures (long-lasting impregnated mosquito nets, LLINs, and intermittent preventative therapy with sulphadoxine-pyrimethamine (IPTp-SP)) among pregnant women in Nigeria.

**Methods:**

Pregnant women selected from among the four communities of Nnewi were interviewed by using a semistructured, interviewer-administered questionnaire on the respondents' demography, knowledge of the cause, consequences, and malaria prevention methods. Also, a total of 48 focused group discussions, 24 key informant interviews, and 24 in-depth interviews were held among women leaders, village heads, pregnant women, community health workers, husbands of pregnant wives, and drug and insecticide-treated net sellers.

**Results:**

A total of 384 women (88.0% third trimester, 90.0% literate, and 41.1% primigravidae) participated. About 80.0% suffered from malaria during their current pregnancy. The majority was aware of the cause of malaria, local name of malaria, mode of transmission, risk of malaria among pregnant women, etc. However, their knowledge and attitude were inadequate regarding the symptomatology and complications of malaria in pregnancy, benefits of sleeping under the net or taking chemoprophylactic doses, or the concurrent use of both. About 80.0% had LLINs, yet only 41.5% slept under it the previous night. Only 31.0% had IPTp-SP doses under direct observation. Only 35.9% had a good understanding of IPTp-SP during pregnancy.

**Conclusion:**

Our work presents important practice gaps associated with the prevention of malaria during pregnancy. The pregnant women seemed to be aware of the basic concepts related to malaria but that does not translate into adequate attitude and practice necessary for malaria reduction.

## 1. Introduction

Worldwide, about three billion people have a risk of contracting malaria, and about 400 million cases and 900,000 deaths from malaria have been seen in recent times [1]. The majority of these appear in sub-Saharan African region [2]. We have five Plasmodium species that are particularly threatening to humans, namely, P. falciparum, P. malariae, P. ovale, P. knowlesi, and P. vivax. The clinical presentation of malaria varies from being mere asymptomatic to symptomatic, affecting different organ systems, depending upon the host's immune system, ecological factors (e.g., temperature, population density, nature of habitats, and feeding habits), and virulence of species [3]. Due to multifactorial contributing conditions, the level of malaria transmission is complicated; for instance, some may develop a degree of immunity to malaria due to rise in exposure times [4]. Although, a huge proportion of population is parasitised worldwide, but the ratio of malaria-related deaths in Africa is touching almost 90% comparatively, especially among children and women [1].

One African country of particular, but unfortunate, interest for malaria is Nigeria, which hosts about 30.0% of the overall malaria burden, while 97.0% of its total population remains at the risk of infection [5]. Moreover, Nigeria suffers an annual economic loss of about 132 billion naira due to malaria [5]. Given such widespread impingement from malaria, the only feasible way to effectively save enormous economic losses and reduce the burden of malaria is through direct community engagement. For instance, health education is seen to improve malaria control outcomes if it addresses the gaps in the knowledge and practice of those who are at risk of infection [6]. Other studies have also supported that people's misconceptions affect malaria control measures [7].

In Nigeria, the National Malaria Control Programme intends to reduce the malaria burden by 50.0% by ensuring at least 80.0% coverage of long-lasting impregnated mosquito nets (LLINs), together with other measures, such as intermittent preventative therapy with sulphadoxine-pyrimethamine (IPTp-SP) for 100% of pregnant women who visit antenatal care (ANC) clinics [8]. However, past studies from Nigeria have reported inadequate expertise and utilization of malaria prevention measures [9]. There also remains a paucity of research, especially with mixed method design [10], about the people's knowledge, attitudes, and practices (KAP) toward malaria in the majority of this extremely large federation.

Thus, with such a vision, we sought to determine the KAP of malaria prevention and management among third trimester and postpartum women. We addressed pregnant women considering their disproportionate risk for contracting malaria [11]. We believe our findings will help to improve the implementation of integrated malaria prevention and also better participation of targeted communities in the control strategies in Nigeria and globally, which is essential for the success and sustainability of disease control programmes [12].

## 2. Methods

The study was conducted among pregnant women, who are in their third trimester or have recently (before discharge from the facility) delivered a child. The women were required to be above 15 years of age, resident (for at least six continuous months), and interested in participation. The study sites were selected from among the four communities of Nnewi, wherein a list of all public and private healthcare facilities was prepared, who were independently given a simple random number. After that, two facilities were selected from each of the four communities. Similarly, all postpartum women before being discharged and consented to participate were selected for inclusion. A semistructured, interviewer-administered questionnaire was used to collect information on the respondents' demography, knowledge of the cause, consequences, and malaria prevention methods. The sample size was estimated by using 52.4% frequency of pregnant women in our province who sleep under LLINs (NMIS, 2010) and 95.0% confidence level (CI) along with 5.0% precision. The required sample size was estimated to be 383 subjects.

All quantitative data were coded, entered into data analyses software, and double-checked by the authors to ensure accuracy. Data analyses were done by SPSS v16. Descriptive statistics were conducted to summarize the respondents' sociodemographic characteristics as well as those related to their KAP regarding malaria prevention in pregnancy. Data collection was pilot tested before the interview and questionnaire distribution at Edorji because it was excluded as a study site. A total of ten questionnaires were administered to ten third trimester women in this pretesting exercise. After the pretesting, the interviewers discussed the questions according to the responses, and required modifications were made.

Local interviewers were trained on basic malaria knowledge, qualitative and quantitative research methods, and research ethics before data collection. Their training also included a review of the interview guides to prepare them for situations that could arise during data collection. Interviewers worked in teams of four, with one person conducting the interviews, the second person taking notes, the third person responsible for digital audio recording, and the fourth person was responsible for translating English to the local language. The interviews took 60-90 min to complete.

**Figure 1:**
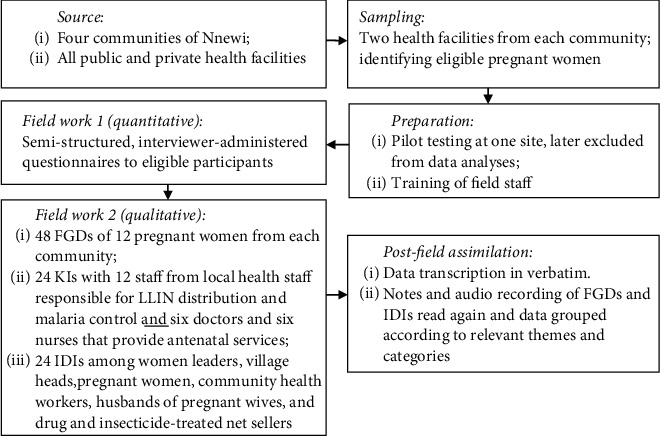
A flow chart depicting the study methods. Notes: FGD: focus group discussions; IDIs: in-depth interviews; LLINs: long-lasting impregnated mosquito nets.

For the qualitative part, a total of 48 focused group discussions (FGDs) were held, each comprising 12 pregnant females from each of the four communities. Twenty-four key informants (KIs) were selected that consisted of 12 staff from the local health department who distribute LLINs and are responsible for malaria control in their concerned sites. The key informants also comprised of six doctors and six nurses from the private and public hospitals that provide antenatal services. A total of 24 in-depth interviews (IDIs) were also held among women leaders, village heads, pregnant women, community health workers, husbands of pregnant wives, and drug and insecticide-treated net sellers. The total number of respondents for the qualitative study was 96 (FGD-48, IDDs-24, and KIs 24). All respondents involved in the KIs were interviewed in their homes or place of work. FGDs and IDIs data were collected through note-taking and audio recording. The data were transcribed verbatim in the participants' own words. The transcriptions and field notes were read over and again, and data were extracted and tabulated according to relevant themes and categories. The study was approved by the Nnamdi Azikiwe University Teaching Hospital Ethics Committee. The subjects were required to provide their written (signature or thumbprint) informed consent after explaining to them the aim of this study. Our study methods have been summarized in a flow chart Figure 1.

## 3. Results

A total of 384 women took part in the study. Their mean age was 28.9 years (95% CI 23.4-34.5). Of them, the majority (*n* = 338, 88.0%) was in their third trimester, while 46 (12.0%) were postpartum before discharge. Of these women, 158 (41.1%) were primigravidae, while 109 (28.3%) were multigravidae. Ninety percent of our respondents (*n* = 345) were literate while the remaining 10.0% were illiterate. The results are provided in Tables 1 and 2.

### 3.1. The Frequency of Malaria Occurence

About 80.0% of our women reported that they suffered from malaria attack during their current pregnancy (Table 2).

### 3.2. The Respondents' Knowledge and Attitude on Malaria and Its Prevention

The majority of respondents were aware of the cause of malaria, a local name of malaria, mode of transmission, disproportionate risk of malaria among pregnant women, the preventability of malaria in pregnancy, the difference between ordinary and treated nets, etc. (Table 1). However, their knowledge and attitude were largely inadequate when it came to the symptomatology of malaria, complications of malaria in pregnancy, benefits of sleeping under the net, benefits of taking chemoprophylactic doses, or the concurrent use of bednets and chemoprophylactic doses (Table 1).

### 3.3. The Practice of Respondents on LLINs

About 80.0% of the women had LLIN in their homes. However, only a fraction of them reported that they usually sleep under the net, and even fewer reported that they slept under the bednet the previous night (Table 2. The reasons for failure to sleep under LLINs nightly were as follows: it feels hot under the net (57.1%), allergic reaction (19.0%), bad odour of the bed net (8.3%), and so on (19.5%).

### 3.4. The Sources for Acquiring the LLINs

Only 4.0% of women had acquired their LLINs from the local shops or the hospital and health centres, while about 40.0% had obtained one during their antenatal visit (Table 2).

### 3.5. The Practice of Respondents on IPTp-SP

The majority of women (39.1%) started taking chemoprophylactic doses during their second trimester, while 13.5% started those before the recommended month (that is, in the first trimester), and the rest began their first dose late in pregnancy. Only 31.0% had taken their required doses under direct observation, while only 35.9% of the women were reminded about their next scheduled uptake of IPTp-SP (Table 2). A total of 44.3% of the women never took IPTp-SP doses in their current pregnancy (Table 2).

### 3.6. The Respondents' Knowledge of ANC Services

The majority of women (60.5%) commenced ANC during second trimester, 25.5% at first trimester while 14.0% began ANC late (i.e., fifth month onwards). About 41.8% had satisfactory (at least seven contacts with ANC providers) ANC attendance (Table 2). Only 76.3% of women had good knowledge regarding the benefits of attending ANCs (Table 1).

### 3.7. Qualitative Responses

“Malaria is called iba in Igboland” (a community leader-IDI 16).

“Malaria is caused by the bite of a female mosquito” (IDI-PW 9).

“Malaria can occur in the environment, when there are bushes around houses, stagnant water in the gutters empty tins and tanks left around the building which can breed mosquitoes which would bite us. If I do not sleep under the treated net, the mosquitos' bite me, and I usually come down with malaria” (FGD-postpartum woman).

“The person will have a headache, a high fever with chills, convulsion, some people will vomit if the condition gets worse” (FGD 13).

“The complications of malaria include maternal anemia, low birth weight, abortion, stillbirth, pre-term delivery” (IDI-PW 11).

“Everybody in the family sleeps under the bed net. If there are a limited number of bed nets, pregnant women and children, under-five should be given priority or individual preference. The consequences of malaria are more in pregnant women than others. So, we should be asked to sleep under the bed-nets more than anyone” (FGDs PW 7).

“Malaria can be prevented by keeping our environment clean, sleeping inside bed nets every night, and by taking three or more dosages of Fansida (SP) at a monthly interval” (FGDs PW 14).

“We were informed of how to hang the net with chemical and to sleep inside the net every night. They also told us to take three white tablets every month after we felt the movement of the baby in the womb” (IDI-Post-natal woman).

“Women who come to this hospital know what causes malaria, the consequences, and how to prevent malaria during pregnancy. I have gained knowledge on how I can prevent malaria by sleeping inside bed net every night as well as drinking the three white tablets of Sulphadoxine-Pyrimethamine every month” (FGD PW).

“Well, we were not told the name of the drug used to prevent malaria during pregnancy neither did we receive health education on it. The nurses do not allow us to know the drug because they remove the drug from its packet. When you come to the hospital, you are told to always take your medicine according to the doctor's prescription. I received three tablets, but I do not know the name of the drug and why I should swallow them and for how long I should take them in this pregnancy” (FGDs PW in private hospital).

“We were not told about the drug called SP” (PW who attends ANC in a private facility).

“We are not told to take SP under their observation; they give drugs in the drug envelope with three tabs starts after a meal” (IDI-PW 19).

## 4. Discussion

Our study was essential since pregnant women are among the most vulnerable population for contracting malaria. Moreover, previous studies have documented a high frequency of malaria throughout Nigeria [13], which was noted in our study as well. This high frequency may likely be due to three times higher general risk of contracting malaria among pregnant than nonpregnant women [11]. Different studies have given different frequencies of malaria for pregnant women in Nigeria and Africa in general [14, 15]. Such differences should be attributed to different geographical location, ecological factors, antenatal attendance, inclusion criteria (e.g., including symptomatic and/or asymptomatic pregnant women), seasonality, etc. For example, studies in Nigeria that had high rates of indoor residual spraying and bednet coverage yield lower frequency of malaria [14]. In contrast, our study had significant poor population-level practices, e.g., nearly 80.0% had LLINs but only about 41.0% had slept under it the last night (Table 1).

Like other African studies [16], our subjects were adequately aware about the general aspects of malaria and its prevention (Table 1), such as having heard about malaria, heard about treated bednets, the local name for malaria, higher risk of malaria during pregnancy, mosquito bite as the mode of malaria transmission, etc. Other studies have shown that knowledge is an independent predictor for the uptake of malaria prevention measures [17]. However, is knowledge adequate to translate into, or ensure, adequate attitude and practice among pregnant women? For instance, 88.6% knew that pregnant women are likely to suffer from malaria more (Table 1), yet only a few of them took their malaria prevention measures (Table 2). These results imply that awareness of malaria may not automatically translate into adequate attitude and practice against malaria but may rather reflect on how individuals perceive various circumstances of using prevention measures and reasons to prevent mosquito bite [18]. For instance, “The first time I took the SP, I did not like the taste” (IDI-PW). Thus, Nigerian authorities must work to bring changes in women's behaviour, such as through behaviour-modifying educational strategies.

LLINs are widely regarded as one of the primary and cost-effective mechanisms for malaria prevention. Nigeria has set a target of 80.0% coverage to LLINs [8], but, plenty remains to be understood and corrected before bednets could practically become an effective and functional antimalaria measure. For instance, 97.0% of our sample had heard about LLINs, and 93.0% understood the difference between ordinary and treated bednets, yet only 59.3% were convinced about the benefits of sleeping under the net (Table 1). Similarly, about 80.0% had LLINs; however, only a small fraction of them sleep under the net regularly, and even fewer had slept under it the previous night (Table 2). Moreover, only 61.3% were convinced about the benefits of using LLINs and chemoprophylaxis concurrently (Table 2). In general, these results are not different from other African studies [19], but specifically, the frequency of daily compliance to LLINs may vary for innumerable reasons between different populations and is most likely context specific. For instance, some African studies show stigmatizing (e.g., bednet as dead body cover) and practical (e.g., inadequate bednet for entire household) patterns related to bednet use [20].

In our study, we identified structural reasons for noncompliance to LLINs (Table 2), for instance, bad odour, heat, and allergic reactions [21]. Other African studies have also shown being inconvenient due to heat and sweating, causing suffocation [22]. Most bednets are made of innately warm materials (e.g., polyester), which makes sleeping under them, even more, warmer in an already hot, humid, and tropical climate. Moreover, additional heat may get generated while reacting with sweat. Thus, Nigerian authorities must focus to identify aerated netting material with superior thermal properties (e.g., ceramic fibre) have a better bednet design with dedicated ventilation pockets or use polymer fibre filament yarns for bednet manufacturing, etc. [23].

As shown in the results section above, there were considerable gaps in the uptake of malaria prophylactic doses. For instance, about 45.0% women never took required doses in their current pregnancy (Table 2). Direct supervised uptake of prophylactic doses is crucial for effective malaria reduction, but its implementation is often a challenge in most malaria settings [24]. Direct observation is useful since women may have their own ideas about the risks and benefits of using IPTp-SP, which may affect their decision to take or not to take necessary doses as required [7] (Table 1). For instance, in Africa, pregnant women are less likely to uptake adequate IPTp doses [25] and may even intentionally avoid taking them up [26]. Moreover, some women may overestimate the risks associated with drug use during pregnancy [27, 28], as seen in Nigeria and elsewhere in Africa. Also, physiological changes due to pregnancy may create a general aversion to oral medications among women [29], which may subsequently challenge malaria prevention in unsupervised circumstances. As in our study (Table 2), others have also shown that some pregnant women receive their medication but do not ingest those [27, 30].

Other African studies also corroborate that high levels of IPTp uptake [31] are attributed to the policy of direct observed therapy, but, if implemented uniformly. Unlike current approach of facility-based supervised voluntary disbursal of oral medications, strategies such as home-based regular domestic health visiting [32] (either alone or in integrated form) may serve several purposes at-once for Nigerian authorities, including the timely delivery of prophylactic medicines, structured follow-ups, and consistent amelioration of knowledge and attitude among recipients in the convenience of recipient's home [33].

Although, training was not a direct outcome of our study, the results of our study show that pregnant women start their chemoprophylaxis late, infrequently take their doses under observation or reminded about them, and more than 2/3 women never took their required doses during current pregnancy (Table 2). We believe that training healthcare staff may help them to better address women's concerns regarding medicational side-effects, communicate with pregnant women using simplified messages [34], and ensure homogeneity in malaria elimination services. For instance, “We were not told about the drug called SP” (PW who attends ANC in a private facility). Elsewhere, it is seen that the practice of IPTp-SP is greatly influenced by the healthcare staff's knowledge of IPTp policy [35]. Other African studies have also shown an increase in the uptake of chemoprohylaxis after the healthcare staff are trained. Thus, service providers are currently a negative factor for inadequate information dissemination to public [36]. Thus, Nigerian authorities need to strengthen their malaria prevention measures through structured training of healthcare workers [37]. Besides staff training, pharmacovigilance studies on IPTp in specific African populations [28] may help to strengthen public's confidence and acceptance on antimalaria doses [38].

In our study, the majority of women (60.5%) commenced ANC during second trimester, while only 42.0% had satisfactory ANC attendance (Table 2). These are important findings since late booking and inadequate ANC attendance by pregnant women affect timely uptake of malaria prevention measures [39], which, in turn, may increase both the risk and frequency of malaria for the population. Other African studies have also shown that malaria prophylactic interventions like IpTP are entirely dependent on ANC platform, and ANC is closely linked to increased malaria case-burden [40]. Thus, Nigerian authorities need to strengthen their ANC platform to solidify their antimalaria measures. For instance, as shown in the results section, only about 40.0% women obtained their LLINs during an antenatal visit.

Lastly, our study had an adequate sample size and was derived from the population. Thus, our results may generalize to the population of our study area as well as elsewhere where similar sociodemographic profiles and health system challenges exist. Moreover, as is with other published reports of similar study designs, our work is also based on self-reports. Nevertheless, our results match fairly well with the general understanding of malaria in our country and in our region.

## 5. Conclusions

In our study, ANC was a source for procuring LLINs for about 40.0% of women; thus, strengthening ANCs may additionally help to strengthen accessibility of LLINs and malaria. Additionally, studying better the safety of IPTp-SP in Africa [28] would further help towards establish adequate acceptance of prevention measures among women [38].

## Figures and Tables

**Table 1:** Malaria prevention knowledge and attitude among the pregnant women in Nigeria.

Items	Frequency (*n*, %)
Heard about malaria	374, 97.3%
Know the local name for malaria in this community	358, 92.5%
Know the mode of transmission of malaria	342, 88.4%
Know the symptoms of malaria	257, 66.4%
Know the complications of malaria in pregnancy	157, 40.6%
Risk of malaria higher among pregnant women	342, 88.6%
Can malaria be prevented in pregnancy?	264, 94.1%
Heard about insecticide-treated net	362, 93.5%
Know the difference between LLIN and ordinary net	358, 92.5%
Know the advantages of sleeping under the net	228, 59.3%
Know the reason for SP administration	183, 47.3%
Know the WHO's recommendations on malaria treatment in pregnancy	131, 33.9%
Need to use SP and LLIN concurrently	228, 61.3%
Benefits of administering three or more doses of SP	193, 49.9%
Essential for pregnant women to go to ANC	367, 94.8%
Know the benefits of ANC	295, 76.3%

Notes: ANC: antenatal care visit; LLIN: long-lasting insecticidal net.

**Table 2:** Malaria prevention practice among the pregnant women in Nigeria.

Item	Frequency (*n*, %)	
*LLIN experience*		
Had LLIN	306, 79.7%	
Were you instructed on how to use LLINs when acquired?	250, 65.1%	
Slept under LLIN the last night	160, 41.5%	
If you had LLIN, where did you purchase? (*n* = 306)	ANC visit	117, 39.7%
	Local shops and H/HCs	12, 4.0%
	Do not know	172, 56.2%
	Others	5, 1.7%
The reasons for not sleeping in the net (*n* = 224)	Feels hot inside	128, 57.1%
	Make me sneeze	39, 19.0%
	Hate the smell	17, 8.3%
	Others	40, 19.5%

*SP experience*		
Received SP in the current pregnancy	214, 55.7%	
Took SP under DOT	119, 31.0%	
Reminded to return for the next SP dose	138, 35.9%	
Taken three SP doses in the current pregnancy	45, 11.6%	
At what month of your pregnancy did you swallow the first dose of SP?	1-3 mnth	52, 13.5%
	4-6 mnth	150, 39.1%
	7-9 mnth	6, 1.5%
	Cannot remember	6, 1.5%
	Did not use SP	170, 44.3%

*Malaria experience*			
If one suffered from malaria in the current pregnancy	Yes	304, 79.2%	
	No	73, 18.9%	
	Do not know	7, 1.8%	

*ANC experience*			
ANC started during	1-2 mnth	99, 25.5%	
	3-4 mnth	232, 60.5%	
	5-6 mnth	45, 11.8%	
	7-8 mnth	5, 1.3%	
	9 mnth	3, 0.9%	
No. of ANC attended	1-2	82, 21.3%	
	3-4	61, 15.8%	
	5-6	81, 21.1%	
	7-8	68, 17.7%	
	More than eight times	92, 24.0%	

Notes: AM: antimalarial medications; ANC: antenatal care visit; H/HC: hospital/health center; LLIN: long-lasting impregnated mosquito nets; mnth: month; SP: sulphadoxine-pyrimethamine.

## Data Availability

All data have been summarized in the manuscript.
